# *GhLBDs* Promote Callus Initiation and Act as Selectable Markers to Increase Transformation Efficiency

**DOI:** 10.3389/fpls.2022.861706

**Published:** 2022-03-25

**Authors:** Ye Wang, Jiachen Yuan, Xi Wei, Yanli Chen, Quanjia Chen, Xiaoyang Ge

**Affiliations:** ^1^Engineering Research Centre of Cotton, Ministry of Education, College of Agriculture, Xinjiang Agricultural University, Ürümqi, China; ^2^Zhengzhou Research Base, State Key Laboratory of Cotton Biology, Zhengzhou University, Zhengzhou, China; ^3^Research Base of State Key Laboratory of Cotton Biology, Henan Normal University, Xinxiang, China

**Keywords:** *Gossypium hirsutum* L., *GhLBDs*, callus, auxin, transformation efficiency

## Abstract

Detached organs or differentiated tissues could form a mass of pluripotent cells termed as callus on an auxin-rich medium, the underlying molecular mechanism of which remains elusive in cotton. LATERAL ORGAN BOUNDARIES DOMAIN (LBD) transcription factor is a key regulator of plant cell totipotency/pluripotency, and a number of cotton *GhLBDs* with high-level differential expression during the callus induction process have been identified. Their overexpression in cotton calli fostered promotions in and callus induction without exogenous auxin. Expression analysis and histological observation using paraffin sectioning suggested that the first 72 h on culture is a key time point for callus initiation, whereby the *GhLBDs* showed high transcript abundance and enlarged calli that were rapidly developed from procambium and cambium. *GhLBDs’* expression level could be precisely modulated by the gradient concentrations of exogenous auxin, whereas auxin transport inhibitor 2,3,5-triiodobenzoic acid could severely inhibit its expression. The LBD-mediated callus formation was also dependent on the expression levels of *GhLBDs*. Further, a β-estradiol-inducible promoter *pER8* was used to drive *GhLBD18-1* expression, which led to rapid callus proliferation, suggesting that *pER8*/*GhLBD18-1* could be used as a selectable marker system to replace the existing antibiotic/herbicide-resistance selectable markers in plant transformation. Our study provides new insights for callus initiation regulatory mechanism and strategies for improving transformation efficiency in cotton.

## Introduction

Plant cells are normally considered as pluripotent cells as most of the differentiated tissues or organs have the potential to regenerate new organs or even whole plants under appropriate culture conditions (Reinert and Backs, 1968; Thorpe, 2007). Plant cell totipotency was one of the top 25 challenging scientific questions by *Science*, and cell totipotency is the cellular basis for plant regeneration (Vogel, 2005). However, the molecular mechanism of plant somatic cell fate reprogramming into pluripotent cells is still unclear, and mining hub genes that regulate the transition of somatic cell to pluripotent cell will optimize the plant transformation system and speed up the regeneration process.

Efficient gene transformation technology has been well established in a number of model plants, such as *Arabidopsis* and tobacco. However, for most crop plants with significant economic importance, such as soybean, cotton, and wheat, it is still in the early infancy stage of development (Anjanappa and Gruissem, 2021). The process of an efficient plant genetic transformation entails somatic embryogenesis (SE) or organogenesis, the essence of which is that somatic cells acquire totipotency through cellular dedifferentiation and redifferentiation. SE is an *in vitro* plant tissue culture process that involves callus initiation, embryogenic callus formation, somatic embryo formation, and plant establishment upon the germination of somatic embryos. In contrast, organogenesis process does not normally involve somatic embryos, and comprises callus induction, shoot induction, and plantlet generation. A common feature of SE and organogenesis is the induction of callus formation from the plant tissue cultured *in vitro*, which remains as a key factor in determining the cell fate transition under conducive conditions for plant regeneration. Callus induction, as the initial step in most *in vitro* plant regeneration system, is a process that differentiated cells dedifferentiate to acquire pluripotency (Cary et al., 2002; Che et al., 2002; Ping and Howell, 2007). Profound molecular genetic changes occur at both the transcriptional and translational levels during the callus induction, especially the genes involved in auxin and cytokinin biosynthesis, metabolism, and signaling pathway (Che et al., 2002; Chitteti et al., 2008; Yang et al., 2012). Biochemical regulation by auxin and cytokinin during plant SE has been well documented, whereby the biosynthesis, accumulation, action, and transport of a number of auxins and cytokinins have been found to work in concert and play a vital role in callus initiation and subsequent progression into embryogenic phase in *Arabidopsis thaliana* and *Brassica napus* (Huang et al., 2014; Rodríguez-Sanz et al., 2015). The intricate balance and an optimal ratio of auxin/cytokinin induce the formation of callus from the explants and promote the acquisition of cell totipotency, which varies from species to species (Skoog and Miller, 1957; Fan et al., 2012).

Cell dedifferentiation is a sophisticated biological process that the extensive modulation of gene expression by auxin/cytokinin underlies dynamic cellular changes. Activation of *WUSCHEL RELATED HOMEOBOX 11* (*WOX11*) and *WOX12* by auxin was necessary for the first step of cell fate transition from the regeneration-competent cell to founder cell, which in turn resulted in the activation of the expressions of *WOX5/7* and *LATERAL ORGAN BOUNDARIES DOMAIN 16* (*LBD16*) as the second step of cell fate transition, from the founder cell to the newly formed callus with cell divisions (Liu et al., 2014; Hu and Xu, 2016; Sheng et al., 2017). *WOX11-LBD16* pathway fosters pluripotency acquisition in callus cells, and its downregulation led to the loss of pluripotency in callus, resulting in shooting defects (Liu et al., 2018). LBDs are dual functional in controlling lateral root (LR) development and callus formation. While *LBD16* and *LBD18* were reported to be responsive to auxin response factors *ARF7* and *ARF19* in controlling LR development (Lee et al., 2009), they were rapidly induced, together with *LBD17* and *LBD29*, by auxin on callus induction medium (CIM); and the ectopic expression of each of the four *LBD* genes was sufficient to trigger callus formation independent of exogenous phytohormones (Fan et al., 2012). Callus initiation followed by the LR pathway, and the establishment of the root primordium-like structure, all mediated by *LBD*s, are the innate features of pluripotency in callus cells (Hu et al., 2017; Liu et al., 2018). Homodimerization and heterodimerization in *LBD* transcription factors are known to be crucial for displaying their biological functions (Lee et al., 2017). Although the four *LBDs* have been demonstrated to be important in the LR development and callus initiation of organogenesis in *Arabidopsis*, their potential role in callus initiation and pluripotency acquisition in SE process is intriguing, but remains elusive.

Cotton is a dual functional crop, proving the world with most plant-derived fiber, and nutritious seeds for oil extraction and use as animal feedstock. The establishment of a highly effective gene transformation system in cotton is hence long overdue as it is a prerequisite for the genetic improvements for such an important economic crop in the world. Distinct from *Arabidopsis* and tobacco among others that could be regenerated by induced organogenesis of callus culture, cotton regeneration occurs mainly *via* SE at considerably low efficiency, which is not only time-consuming but also only feasible in a few agronomically irrelevant genotypes. Given that dedifferentiation is the first key step in the whole process of SE that somatic cell dedifferentiation directly determines the ability of callus cell acquiring pluripotency, hub genes mining from the differentially expressed genes in callus initiation process may provide a useful approach for identification of key molecular elements that govern the intricate modulation of SE process. In this study, we describe our attempts in using such a research strategy in the investigation leading to the identification of LBD transcription factors that mediate the callus induction prior to SE. We unraveled their expression profiles in the callus initiation and LR formation stages, as well as their inducibility by auxin. We have also found that overexpression of the *LBD* genes promotes callus formation in the absence of exogenous auxin; and the induction of *LBD* expression by β-estradiol could be used as a visible and reliable marker for embryogenic calli, which may prove to be useful in improving the efficiency of cotton transformation.

## Materials and Methods

### Plant Material, Growth Conditions, and Plant Transformation

Upland cotton (*Gossypium hirsutum* L.) cultivar CRI24 that is relatively amendable for regeneration was used in this study. Cotton seedlings derived from the germination of surface-sterilized cottonseeds on MS medium were maintained in a growth chamber with a controlled environment for 7 days, with a 14/10 h photoperiod at 28°C. As previously described (Ge et al., 2015), the hypocotyls excised from the aseptic seedlings were inoculated with *Agrobacterium tumefaciens* strain LBA4404 cells harboring the *35S*::GhLBDs or *pER8*:GhLBD18-1 plasmids. Upon the induction of calli, somatic embryos were generated and developed into plantlets. All the explants, calli, somatic embryos, and regenerated seedlings were maintained in a tissue culture room at 25–28°C under a 16 h light/8 h dark photoperiod.

### RNA Extraction, Gene Cloning, and Vector Construction

Total RNA was isolated from 7-day-old sterile seedlings of *G. hirsutum* CRI24 plants using the Plant Total RNA Extraction Kit (TIANGEN Biotech, Beijing, China). The cDNA was synthesized using a PrimeScript RT reagent kit with gDNA eraser (Takara, Dalian, China). Primers (Supplementary Table 1) for *GhLBD*s were designed using Premier Primer 5 and used to amplify the full-length coding sequences of *GhLBDs*. The amplified products were each linked to the pBI121 expression vector under the transcriptional control of *35S* CaMV promoter using the proper restriction endonuclease. The constructed vectors were introduced into LBA4404 strain, and positive clones were selected to transform the cotton hypocotyls with kanamycin as the selectable agent.

### Expression-Level Analysis by Quantitative Real-Time Reverse Transcription Polymerase

For expression analysis of *GhLBDs*, LRs, primary roots (PRs), and explants at different time points in the early stage of cotton callus formation were collected, from which total RNA and cDNA were prepared as described above. For quantitative real-time reverse transcription polymerase (RT-PCR), endogenous gene *GhHistone 3* (Gh_D03G0370) was used as an internal reference gene. The quantitative RT-PCR was performed on the Applied Biosystems 7900HT system (Thermo Fisher Scientific, Waltham, MA, United States) using ChamQ Universal SYBR qPCR Master Mix (Vazyme Biotech, Nanjing, China). CT-method (2^–ΔΔCt^) was used to quantify the relative expression level of the target genes. All the primer sequences are given in Supplementary Table 1.

### Auxin Indole-3-Acetic Acid and Auxin Transport Inhibitor 2,3,5-Triiodobenzoic Acid

Callus induction medium was supplemented with 2,3,5-triiodobenzoic acid (TIBA) or Indole-3-acetic acid (IAA)(2,4-D). To test the dose effects of auxin on the induction of *LBD* expression, a range of four different concentrations of TIBA, 0, 10, 20, and 30 μM, and a range of five different concentrations of 2,4-D, 0, 0.1, 0.3, 0.4, and 0.5 mM were used.

### Histological Observation and Scanning Electron Microscopy

The hypocotyls of 7-day-old sterile seedlings of overexpressing *GhLBDs*, together with those of the negative control lines, were cut into segments of 1 cm in length and placed on CIM for 0–120 h prior to fixation in 50% FAA (50% ethanol, 10% formalin, 5% acetic acid, and 35% water) overnight. The fixed tissues were then embedded in paraffin prior to sectioning and microscopy observation previously described (Alexander et al., 2018). Selected calli were harvested following their culture on CIM medium for a period of 15, 25, and 45 days and prepared for the analysis of cell morphology under a HITACHI SU3500 scanning electron microscope (Hitachi, Tokyo, Japan).

### Domains Analysis and Phylogenetic Analyses

LBD protein sequences from *A. thaliana* were used as queries for searching the homologs in *G. hirsutum* database using BlastP program, and the hits with *e*-values of 1e-5 were considered as significant (Zhang et al., 2015). As a result, 132 candidate *LBD* genes in *G. hirsutum* were identified. MEGA 7.0 was used to construct phylogenetic tree. PROSITE^1^ and InterProScan^2^ were used for domain search.

## Results

### Morphological Characterizes and Cytological Changes in the Formation Process of Callus

**FIGURE 1:**
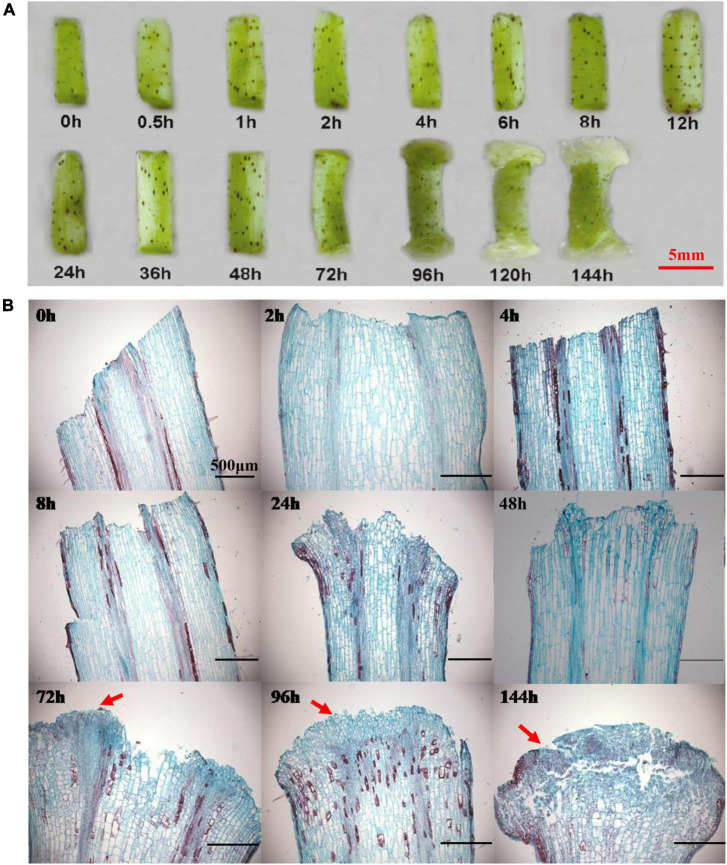
The morphological and cytological changes during the initiation of callus. **(A)** The morphological changes during the callus initiation. **(B)** The cytological changes during the formation of callus. Red arrows indicate the callus initiation. Different time points include 0, 0.5, 1, 2, 4, 6, 8, 12, 24, 36, 48, 72, 96, 120, and 144 h when hypocotyls were placed on callus induction medium. Scale bar: 5 mm **(A)** and 500 μm **(B)**.

Sterile seedling hypocotyls were used as explants to induce callus initiation on CIM. The observation of callus initiation process indicated that there were no obvious morphological and cytological variations during the initial 48 h on CIM (Figure 1), suggesting that this could be a preparation stage for callus induction. The two ends of a hypocotyl became slightly enlarged, and the cell mass from the procambium and cambium began to form on the ends of hypocotyl following 72 h on culture, which marked the initiation of callus. This is congruent with previous studies that the calli of different explants mainly originated from the pericycle cells in the tissue (Ping and Howell, 2007; Atta et al., 2008). The callus cells were then quickly proliferated and became distinctively visible at 96 h (Figures 1A,B). Notably, one end of the hypocotyl produced healthy and large calli, in contrast to the other end with slowly growing and smaller calli in the first 144 h. Such an observation could be attributed to the variable auxin levels at the two ends of a hypocotyl, in which the auxin gradient polarity stemming from the differential distributions of the endogenous auxin had affected the callus initiation and development.

### Identification of *LBD* Genes Preferentially Expressed During Cotton Initiation Process

**FIGURE 2:**
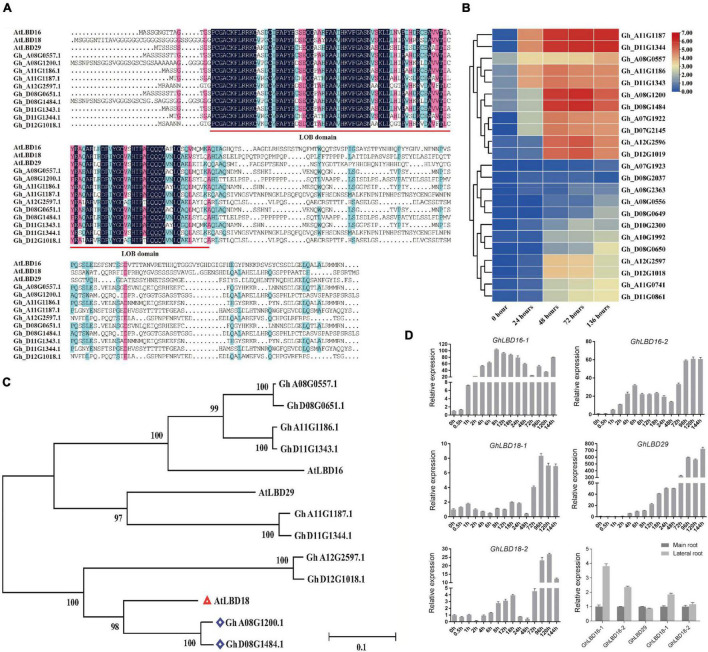
Expression patterns of highly expressed *LBD* genes during callus initiation stage and phylogenetic analysis. **(A)** Alignment of LOB domain analysis between *Gossypium hirsutum* L. and *Arabidopsis*. **(B)** Heat map of subgroup IV genes during callus initiation process (0–136 h). Different time points include 0, 24, 48, 72, and 136 h when hypocotyls were placed on callus induction medium. **(C)** Genetic evolutionary tree analysis of *LBD* genes highly expressed in callus initiation stage in *Gossypium hirsutum* L. and *Arabidopsis*. **(D)** Expression pattern of GhLBD16/18/29 homologous during the callus initiation, main root, and lateral root. Different time points include 0, 0.5, 1, 2, 4, 6, 8, 12, 18, 24, 48, 72, 96, 120, and 144 h when hypocotyls were placed on callus induction medium.

The knockout mutation of the WOX11-LBD16 pathway resulted in a complete loss of pluripotency in the callus of *Arabidopsis* (Liu et al., 2018), suggesting that some specifically expressed *LBD* genes during the cotton callus induction stage may also be crucial for callus proliferation and pluripotency acquisition, in addition to their known role in callus induction. In light of the transcriptome data, we have identified a number of annotated *LBD* genes that presented relatively a higher level of expression during callus initiation stage (Figure 2B), supporting the notion that LBDs play a key role in regulating callus initiation and growth.

It is generally recognized that phylogenetic analyses may enable the revelation of functional redundancy of gene families as the genes belonging to the same subgroup normally have overlapping functions. For further investigation into their potentially diverse or overlapping functions, all the annotated 131 LBDs were used to perform phylogenetic analyses (Supplementary Figure 1). LBDs were divided into eight subgroups, among which the aforementioned LBDs that were highly expressed during callus initiation stage were grouped into subgroup IV (Supplementary Figures 1, 2). By virtue of domain motif analysis, most members the subgroup IV were found to contain motif 8 that is unique to the subgroup, suggestive of functional divergence. This is well in line with a previous study that diversification of regulatory regions led to subfunctionalization or neofunctionalization (Li et al., 2005).

To further determine the evolutionary and homologous relationship, these above highly expressed *GhLBD* genes were used to perform phylogenetic analysis with *Arabidopsis AtLBD*s. As shown in Figure 2C, AtLBD16, AtLBD18, and AtLBD29 were clustered together with the aforementioned LBDs that were highly expressed during callus initiation stage. Based on the phylogenetic analysis, Gh_A11G1187/Gh_D11G1344, Gh_D08G1484/Gh_A08G1200, Gh_A08G0557/Gh_D8G0651, Gh_A11G1186/Gh_D11G1343, and Gh_A12G2597/Gh_D12G1018 were named as GhLBD29, GhLBD18-1, GhLBD16-1, GhLBD16-2, and GhLBD18-2, respectively (Figure 2C). PROSITE (see text footnote 1) and InterProScan (see text footnote 2) were used to search for the LOB domain in the obtained sequences and found that the LOB domain was highly conserved in LBD16, LBD18, and LBD29 between *G*. *hirsutum* and *Arabidopsis* (Figures 2A,C).

### Expression Patterns of *GhLBD16, GhLBD18, GhLBD29* in Lateral Root and Callus Initiation

The regulatory network of controlling LR formation is highly conserved with that of controlling callus formation (Fan et al., 2012; Xu et al., 2018). The comparative expression of *GhLBD16/18/29* homologous between the PR and LR was analyzed using qRT-PCR. As shown in Figure 2D, four out of the five candidates, including *GhLBD16-1/2, GhLBD18-1/2*, and *GhLBD29*, exhibited higher expression levels in LR relative to PR. Considering the similar regulation mechanisms between LR formation and callus development, expression patterns of these *GhLBDs* in the callus initiation process were detected by qRT-PCR (Figure 2D). Significant variation was observed in their expression patterns, including the expression abundance and peak point. The expressions of *GhLBD16-1*, *GhLBD16-2*, and *GhLBD29* were similar, with a rapid response to the induction during the early initiation stage within 24 h. It is envisaged that *GhLBD16* and *GhLBD29* may function in concert by forming homodimers or heterodimers to induce cell proliferation (Lee et al., 2017). In contrast, *GhLBD18-1* and *GhLBD-2* showed relatively low expressions during the callus initiation process of 48 h. Notably, all the five *GhLBDs* showed considerably higher expressions at the late initiation stage from 72 to 144 h in comparison to the early initiation stages. Data shown in Figure 1 also suggested that 72 h might be the key time point for callus initiation and proliferation. The variation in peak time points and expression abundance among these *GhLBDs* are the manifestations of their functional redundancy and potential divergency. Such a premise is supported by the analysis of paraffin sections (Figure 1B) that exhibited conspicuous callus phenotype at 72 h stage.

### The Expression of *GhLBD16/18/29* Homologs and Callus Initiation Was Regulated by Exogenous Auxin Concentration and Auxin Polar Location

**FIGURE 3:**
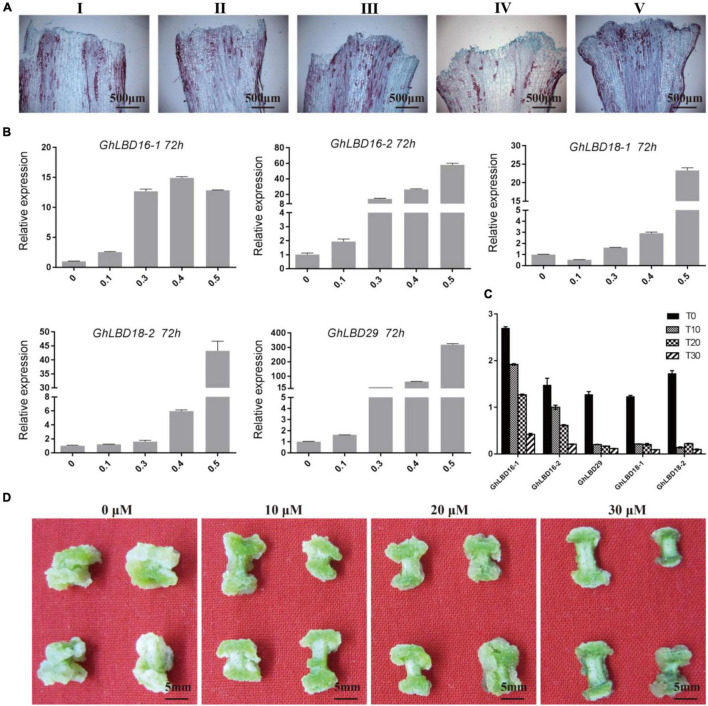
2,4-D and TIBA treatment affect the expression of *GhLBDs* and callus proliferation. **(A)** The phenotype of callus initiation was observed at 72 h after adding gradient concentrations of 2,4-D. I: 0 mM 2,4-D; II: 0.1 mM 2,4-D; III: 0.3 mM 2,4-D; IV: 0.4 mM 2,4-D; and V: 0.5 mM 2,4-D. Scale bar: 500 μm. **(B)** The relative expression level of *GhLBDs* in explants at 72 h after induction by gradient concentration of auxin. **(C)** Expression profile of *GhLBDs* at 72 h with gradient concentration treatments of TIBA. T0, 0 μM TIBA; T10, 10 μM TIBA; T20, 20 μM TIBA; and T30, 30 μM TIBA. **(D)** Callus phenotypes at 30 days after adding gradient concentrations of TIBA. Scale bar: 5 mm.

To investigate *GhLBDs’* response to exogenous auxin, five different concentrations of 2,4-D and three different concentrations of auxin transport inhibitor TIBA were added in the callus-induced medium. After 72 h induction, the expression of *GhLBD16/18/29* was analyzed using qRT-PCR. Five *GhLBDs* genes showed very low expression without exogenous auxin treatment. A significant increase in *GhLBDs’* expression was observed after exogenous auxin treatment (Figure 3B). The paraffin sections derived from the callus samples supplemented with different auxin concentrations indicated that 0.4 mM 2,4-D was the minimal amount to be effective in promoting callus initiation (Figure 3A), significantly enhancing *GhLBDs’* expression at 72 h (Figure 3B). Lower concentrations of auxin with less than 0.4 mM were able to induce slightly, but not sufficient enough in inducing callus initiation at 72 h. TIBA treatment disrupted the localization and movement of auxin through impacting on auxin polar transport protein PINs (Cristian et al., 2010), resulting in the reduction in auxin level at the ends of hypocotyl explants. The low auxin level in turn cannot be sufficient enough in activating the expression of *GhLBDs* and delayed callus initiation at the ends of hypocotyl explants. As is evident in Figures 3C,D, TIBA treatment significantly inhibited the expression of *GhLBDs* and callus proliferation in cotton hypocotyl explants, in agreement with the notion that the auxin polar location is positively associated with *GhLBDs’* expression and callus induction.

### *GhLBD16/18/29* Homologs Have Synergistic Function With Exogenous Auxin in Promoting Callus Growth but Are Independent of Exogenous Auxin

**FIGURE 4:**
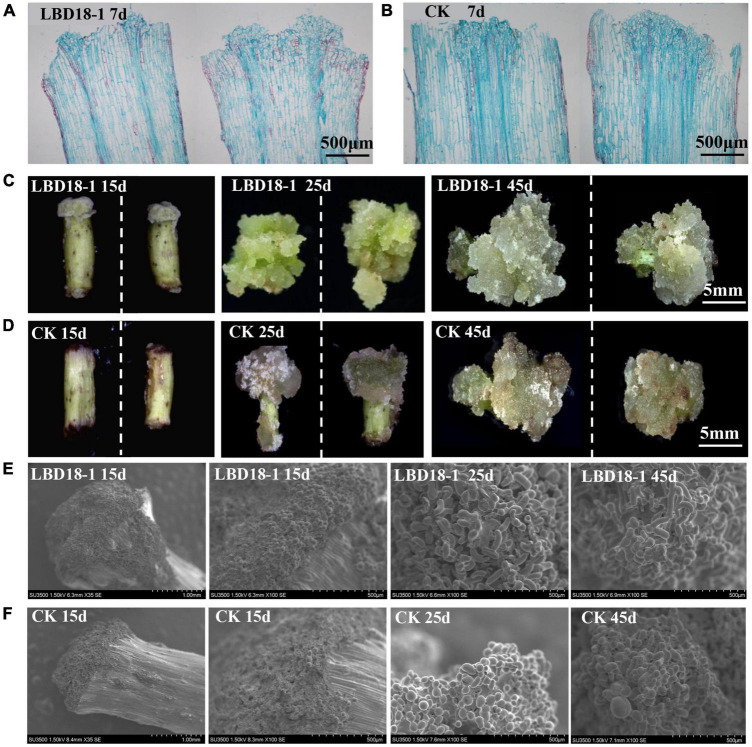
Observation of *GhLBD18-1* transgenic and CK lines induced on CIM medium without exogenous auxin. **(A,B)** Histological observation of *GhLBD18-1* transgenic and CK lines induced on CIM medium without exogenous auxin at 7 days post induction. **(C,D)** The callus phenotypes of transgenic and controls lines after treatment of 15, 25, and 45 days on CIM without exogenous auxin. **(E,F)** SEM observation of *GhLBD18-1* transgenic lines and CK (lines transformed with pBI121 empty vector) at 15, 25, and 45 days post induction. 15d, callus after subculture of 15 days on CIM; 30d, callus induction after subculture of 30 days on CIM; and 45d, callus induction after subculture of 45 days on CIM. **(A,B)** Scale bar, 500 μm; **(C,D)** scale bar, 5 mm; **(E,F)** scale bar, 1 mm and 500 μm.

**FIGURE 5:**
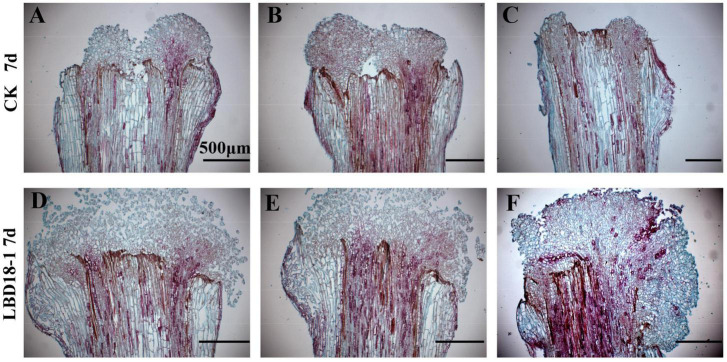
The paraffin section of transformation and empty vector at 7 days on the CIM. **(A–C)** The paraffin section of control hypocotyls after treatment for 7 days on CIM with exogenous auxin. **(D–F)** The paraffin section of transgenic *GhLBD18-1* hypocotyls after treatment for 7 days on CIM with exogenous auxin. Scale bar: 500 μm.

To confirm the functions of *GhLBD16/18/29* homologs, transgenic cotton lines overexpressing *GhLBD16/18/29* under the transcriptional control of *35S* CaMV promoter were generated. To abrogate the confounding effects of auxin induction on *GhLBD16/18/29* expression, the exogenous auxin was omitted from CIM to determine whether the GhLBD-induced callus growth is dependent on exogenous auxin. After subculturing on CIM without exogenous auxin for a period of 7 days, there was no callus formation discernible by naked eyes in both overexpression lines and controls that are transformed with pBI121 empty vector. Cytological examination on paraffin sections found small yet distinctive callus cell masses at the ends of hypocotyls of *GhLBD18-1* transgenic lines, which was in contrast to the control where only the proliferated cells in the procambium and cambium were spotted (Figures 4A,B). This is clearly suggestive of the role of *GhLBD18*-1 in promoting callus initiation without the need for exogenous auxin. Similar observations have also been made with the transgenic lines overexpressing *GhLBD16-1, GhLBD16-2, GhLBD18-2*, or *GhLBD29* (Supplementary Figure 3B). After being subcultured for 15 days on CIM without exogenous auxin, calli of considerable sizes were formed at the ends of hypocotyl explants derived from all the *GhLBDs’* overexpressing lines, but no callus was formed in the control (Figure 4C and Supplementary Figure 4). Notably, both the *GhLBDs* overexpressing lines cultured on CIM without exogenous auxin and the control lines cultured on CIM containing exogenous auxin produced calli in similar sizes after 15 days in culture. At 25 days after culture, abundant calli were displayed in the transgenic lines overexpressing *GhLBD18-1*, in sharp contrast to the controls that only a few tiny calli were discernible. Consistent observations were made at 45 days in culture (Figures 4C,D). The morphology of the callus cells was analyzed using SEM. Despite the size difference (Figures 4C,D), the callus cells were all in globular shape in both *GhLBD18-1* overexpression lines and control plants at 15-day subculture (Figures 4E,F). Rod-shaped cells were observed in *GhLBD18-1* overexpression lines, whereas the control cells remained as globular or became oval shape at 25 and 45 days in subcultures (Figures 4E,F). Given both *GhLBDs’* expression and exogenous auxin could induce callus initiation, the question arises as to whether these two different inducers could operate in a synergistic manner. To address this question, a comparison was made for the calli overexpressing *GhLBD18-1* on cultures with and without exogenous auxin. Following 7 days in culture, the callus mass was substantially larger when exogenous auxin was added to the CIM medium for both transgenic and WT control (Figure 5). Corroborating results were also obtained in the transgenic calli expressing *GhLBD16/18-2/29* (Supplementary Figure 3). Taken together, our results demonstrated that all the tested *GhLBD* genes, including *GhLBD16/18/29*, could not only promote the initiation and growth of callus cells independent of exogenous auxin, but also act synergistically with exogenous auxin in regulating callus proliferation.

### The Expression of *GhLBD18-1* Is Proportional to the Vigor of Callus Growth

**FIGURE 6:**
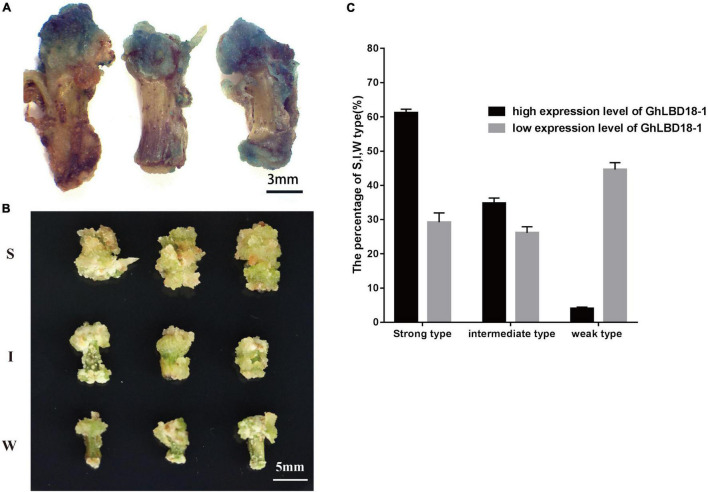
Callus growth vigor was different among highly expressed and lowly expressed *GhLBD18-1* lines. **(A)** GUS staining indicating that *GhLBD18-1* was expressed in callus cell mass. Scale bar: 3 mm. **(B)** S, strong-type callus with large size; I, intermediate-type callus with moderate size; and W, weak-type callus with small size. Scale bar: 5 mm. **(C)** The percentage of S, I, and W callus was calculated between highly expressed and lowly expressed *GhLBD18-1* OE lines. Hypocotyls were cultured to induce callus in CIM without exogenous auxin, and the percentage of S, I, and W types were calculated on the 30th day after subculture. In total, 300 sections of hypocotyls were cultured for *GhLBD18-1* OE lines and control plants, and the experiment was repeated three times.

Transgenic cotton lines overexpressing *GhLBD18-1* were divided into two groups, i.e., high (H-type) and low (L-type), depending on whether the expression of *GhLBD18-1* reached 10-fold of the WT level. As *GhLBD18-1* was fused in frame with the β*-glucuronidase* (*GUS*) gene being controlled by *35S* promoter in pBI121, the transgenic calli were stained blue in color (Figure 6A). According to the growth rate and morphology size of callus, we divided the callus into three types, namely, strong (S, enlarged size), intermediate (I, moderate size), and weak (W, small size) (Figure 6B). The calli derived from the H-type *GhLBD18-1*-overexpressing lines showed active growth and robustness on the CIM without exogenous auxin, in contrast to those of L-type transgenic lines. In addition, the numbers of S and I types of calli derived from the H-type transgenic lines were significantly increased, whereas the number of the W-type calli was obviously reduced (Figure 6C). For the lines transformed with an empty vector, callus initiation and growth were substantially inhibited, whereby most hypocotyl segments failed to produce discernible callus tissues at 30 days on CIM medium without exogenous auxin (Supplementary Figure 5).

### GhLBD18-1 Might Be Used as a Novel Selectable Marker in Cotton Genetic Transformation

**FIGURE 7:**
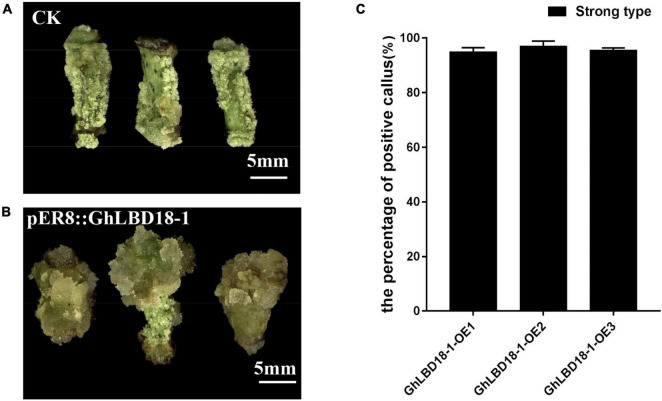
GhLBD18-1 as an alternative selectable marker for genetic transformation. **(A,B)** Negative and positive callus. CK, negative callus; *pER8*::GhLBD18-1, positive callus. Scale bar: 5 mm. **(C)** The percentage of positive callus. S, strong-type callus with large size.

Antibiotic and herbicide resistance genes are commonly used for positive cell selection in plant genetic transformation, but it is highly desired to develop an alternative selection approach in order to abrogate the needs for potentially harmful chemicals. Moreover, antibiotics that are used as selective agents could inhibit cell growth vigor and impose negative effects on cell proliferation and differentiation (Padilla and Burgos, 2010). The overexpression of *GhLBD18-1*, therefore, may represent an alternative selectable marker as it could give the transformed cells an advantage of cell growth over the untransformed cells. The overexpression of *GhLBD18-1* driven by a β-estradiol-inducible promoter *pER8* (Zuo et al., 2000) fostered positive callus cell growth as evidenced by the larger calli than the control calli at 30 days on CIM containing 10 μM β-estradiol (Figures 7A,B). Further examination of the large calli by qRT-PCR showed that more than 95% of them were genuine transgenic (Figure 7C). In essence, these results indicated that the overexpression of *GhLBD18-1*, coupled with β-estradiol as an induction agent, may serve as an effective and novel selection system for gene transformation in cotton.

## Discussion

### GhLBD16/18/29 Function Redundantly in Regulating Callus Initiation and Proliferation

The cotton *GhLBD* gene family is composed of 131 members that could be classified into eight subgroups *via* phylogenetic analysis (Supplementary Figure 1). As cotton is an allotetraploid plant species, many of these genes could be in homeologous pairs (Yang et al., 2019). By virtue of the expression analysis in callus induction stage, five preferentially expressed *GhLBD* genes, including *GhLBD16-1, GhLBD16-2, GhLBD18-1, GhLBD18-2*, and *GhLBD29*, were selected as candidates for study, and their potential functional role was explored in relation to callus induction and callus growth vigor. The five selected *GhLBD* genes were all significantly induced by exogenous auxin or inhibited by auxin transport inhibitor TIBA (Figure 3), suggesting that these GhLBDs may act downstream of auxin regulation and play a similar role in regulating callus formation and development. Differential expression of these *GhLBD* genes was illustrated, whereby *GhLBD16-1, GhLBD16-2*, and *GhLBD29* presented similar expression patterns that are significantly induced in the early initiation stage (1–144 h), whereas *GhLBD18-1* and *GhLBD18-2* expressed rather late, with a relatively high level of expression at 72–144 h following the culture initiation. A similar study also proved that LEAFY is a pioneer transcription factor in promoting floral fate (Jin et al., 2021). fgfbp genes family showed temporal and spatial expression patterns to maintain zebrafish embryo development (Li et al., 2018). Our results are congruent with previous studies that *Arabidopsis* orthologs of *GhLBD16, GhLBD17, GhLBD18*, and *GhLBD29* functioned redundantly in triggering callus formation (Fan et al., 2012). Because these *GhLBD* genes were grouped in the same clade in phylogenetic analysis (Supplementary Figures 1, 2), functional overlapping or divergency to some extent could be anticipated, as elucidated in previous studies (Mangeon et al., 2012). Overexpression of each of the five *GhLBD* genes in cotton was sufficient to trigger callus formation without exogenous auxin (Figure 4 and Supplementary Figures 3–5), suggestive of functional redundancy, but detailed examination on their individual role in modulating callus initiation and proliferation requires further exploration.

### Callus Growth Vigor Is Dose-Dependent on the Expression Level of *GhLBD18-1*

Gene expression is often closely related to its function and is commonly used to infer functionality, although it may not be directly proportional. Appropriate upregulation of *BOC1* expression could reduce callus browning in rice, both high and low expressions of BOC1 led to serious callus browning and increased ratio of browning callus (Zhang et al., 2020). In transgenic cotton plants overexpressing *GhLBD18-1*, callus size and growth vigor were found to have a direct positive correlation with transgene expression level, whereby about 60 vs. 29% large callus, and about 35 vs. 26% moderate-sized callus in highly expressed *GhLBD18-1* lines and moderately expressed *GhLBD18-1* line, respectively (Figure 6). Altogether, these results indicated that *GhLBD18-1* expression promotes callus growth in a dose-dependent manner. However, excessive callus proliferation could be unfavorable for cell pluripotency acquisition, and appropriate level of *GhLBD18-1* expression may be required to ensure the optimal callus proliferation and cell fate transition to embryogenic callus.

### *pER8*::LBD18-1 Is a Promising Transgenic Selection System

The promotion of callus initiation and proliferation independent of exogenous auxin by overexpressing *GhLBD* in cotton suggested their functional role downstream of auxin regulation for callus growth. To avoid the potential pleiotropic effects and precise modulation of callus growth, a β-estradiol-inducible promoter *pER8* was employed to upregulate *GhLBD18-1* expression when it is required. It is also known to have moderate strength relative to *35S* CaMV promoter (Schlücking et al., 2013). Furthermore, we have also examined the feasibility of using *GhLBD18-1* as a selectable marker to substitute for the currently used antibiotics or herbicide-resistance marker gene. The abrogation of antibiotic and herbicide screening would enable calli to thrive on CIM without chemical stress. Moreover, the use of antibiotics or herbicide resistance in screening for transgenic plants is accompanied by the frequent escape of non-transgenic cells that may form a chimeric tissue with transgenic cells, causing undesirable complications in the selection and lowering transgenic efficacy. Furthermore, there are ever-increasing public concerns for the unintended gene flow of chemical resistance into the environment, which also results in the heavy regulatory burden and prolonged delays in releasing transgenic crops. Therefore, the employment of *GhLBD18-1* coupled with *pER8* has clear advantages as a promising transgenic selection system in plant genetic transformation relative to the classic antibiotic/herbicide-based selection systems.

## Data Availability Statement

The datasets presented in this study can be found in online repositories. The names of the repository/repositories and accession number(s) can be found in the article/Supplementary Material.

## Author Contributions

QC and XG conceived and instructed the study. YW performed the main experiments. JY participated in the experiments and performed the data analysis. XW and YC bred the plant materials. YW and XG wrote the manuscript. All authors contributed to manuscript revision, read, and approved the submitted version.

## Conflict of Interest

The authors declare that the research was conducted in the absence of any commercial or financial relationships that could be construed as a potential conflict of interest.

## Publisher’s Note

All claims expressed in this article are solely those of the authors and do not necessarily represent those of their affiliated organizations, or those of the publisher, the editors and the reviewers. Any product that may be evaluated in this article, or claim that may be made by its manufacturer, is not guaranteed or endorsed by the publisher.

## Footnotes

1
http://prosite.expasy.org/

2
http://www.ebi.ac.uk/interpro/
