# Supplementary material for: GhLBDs Promote Callus Initiation and Act as Selectable Markers to Increase Transformation Efficiency
Source: Front Plant Sci. 2022 Mar 25;13:861706. doi: 10.3389/fpls.2022.861706 (PMC8990305; doi:10.3389/fpls.2022.861706)
Supplement: Supplementary file 1 [file Data_Sheet_1.docx]

*GhLBDs* promote callus initiation and act as selectable markers to increase transformation efficiency

**Ye Wang^1,3#^, Jiachen Yuan^2#^, Xi Wei^3#^, Yanli Chen^2^*, Quanjia Chen^1*^, Xiaoyang Ge^1,2,3*^**

^1^Engineering Research Centre of Cotton, Ministry of Education / College of Agriculture, Xinjiang Agricultural University, 311 Nongda East Road, Urumqi, 830052, China.

^2^Zhengzhou Research Base, State Key Laboratory of Cotton Biology, Zhengzhou University, Zhengzhou 450001, China.

^3^Henan Normal University Research Base of State Key Laboratory of Cotton Biology, Xinxiang 453000, China.

^#^ These authors contribute equally to the present work.

***Correspondence**

Yanli Chen

cylxr2012@163.com

Quanjia Chen

chqjia@126.com

Xiaoyang Ge

gexiaoyang@caas.cn

Supplementary Material


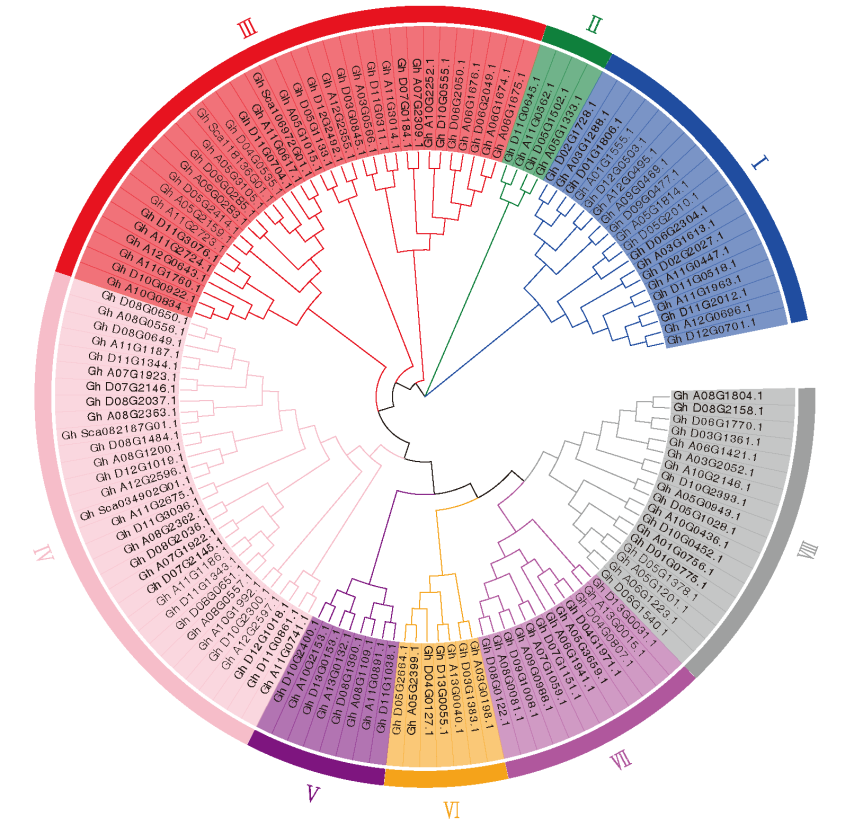


**Supplementary Figure 1 Phylogenetic analyses showed that *GhLBDs* were divided into eight sub-groups.**


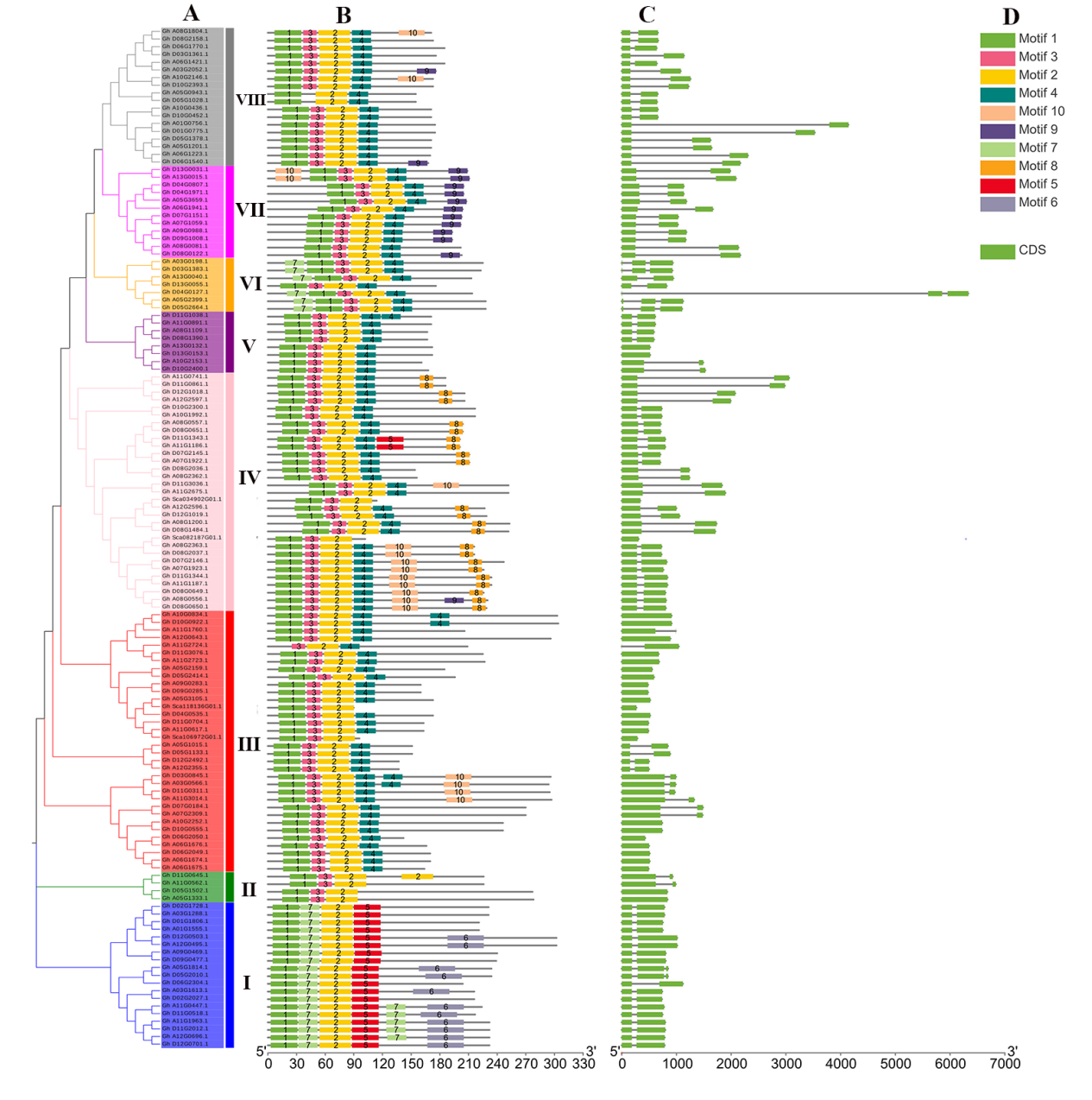


**Supplementary Figure 2 Based on the domain motif analysis, most members in sub-group IV contained the unique motif 8.**


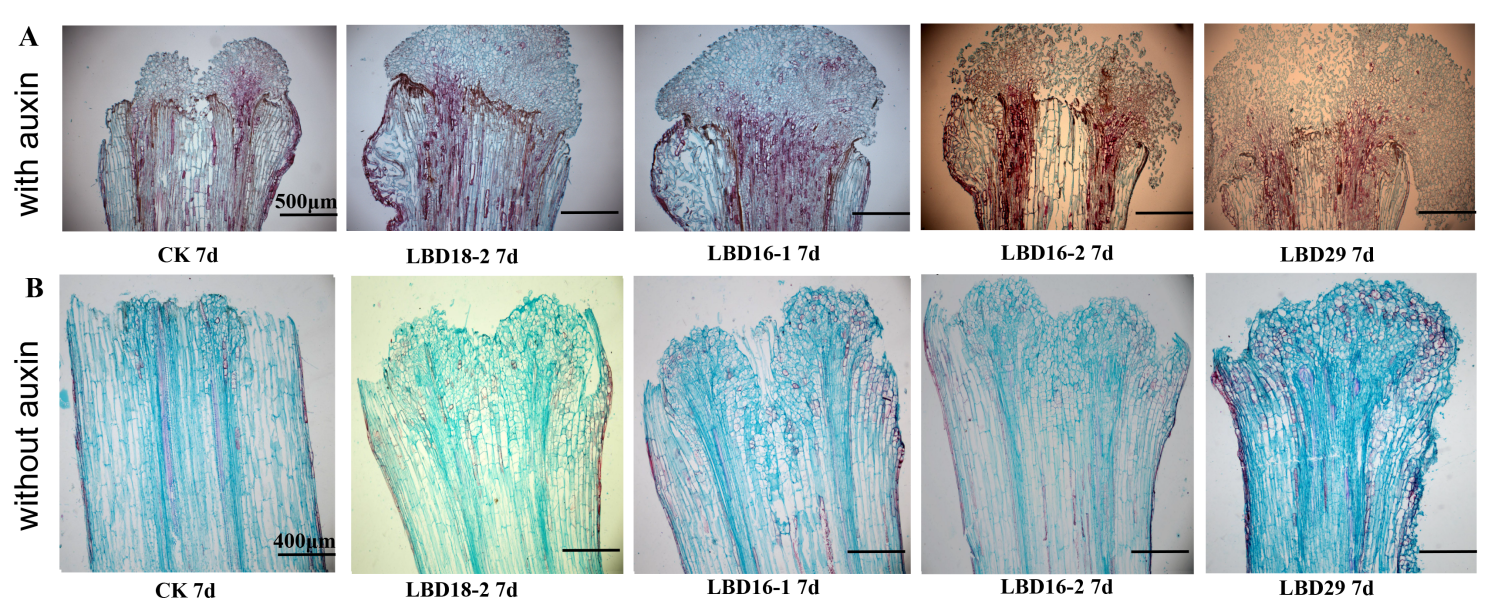


**Supplementary Figure 3** **The paraffin section of transformation and empty vector hypocotyls after treatment of 7d on the CIM with(A) and without(B) exogenous auxin.**


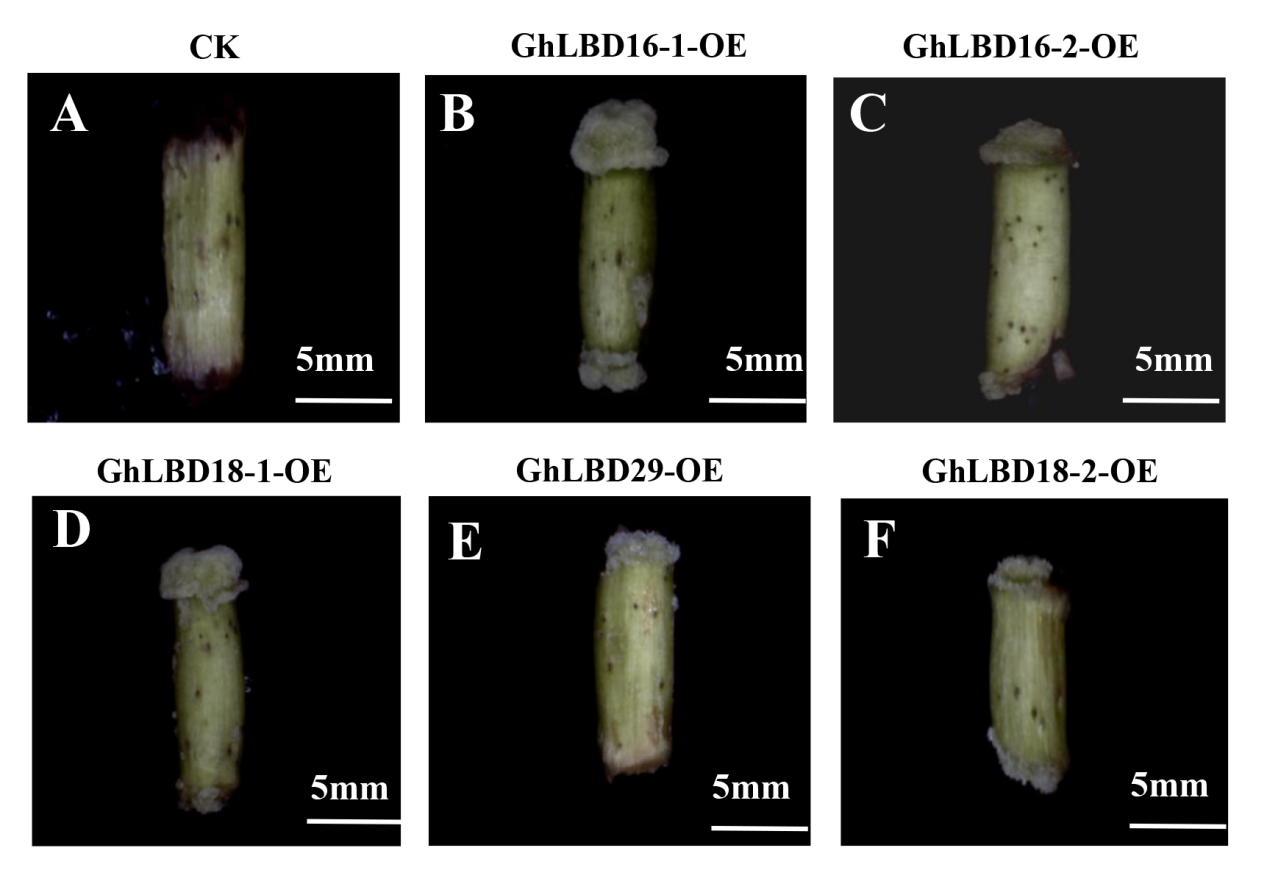


**Supplementary Figure 4** **Observation of *GhLBD16/18/29* transgenic and CK lines after treatment of 15 d on the CIM medium without exogenous auxin.** Scale bar, 5mm.


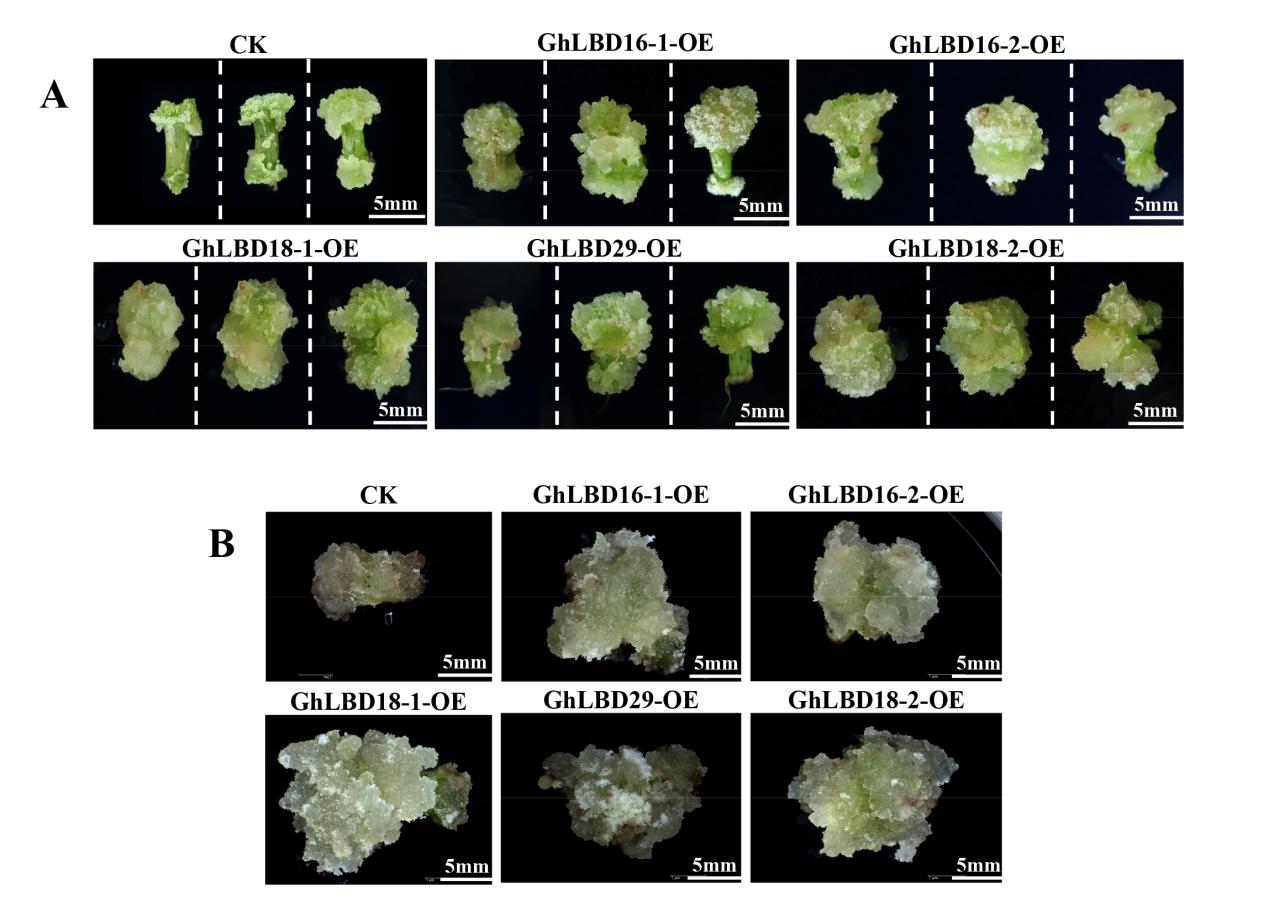


**Supplementary Figure 5 Observation of *GhLBD16/18/29* transgenic and CK lines after treatment of 30 and 45 d on the CIM medium without exogenous auxin.** (A) The callus phenotypes of transgenic and controls lines after treatment of 30 d on CIM without exogenous auxin. Scale bar,5mm. (B)The callus phenotypes of transgenic and controls lines after treatment of 45 d on CIM without exogenous auxin. Scale bar, 5mm.

**Supplementary table S1**

| **Primer name** | **Sequences (5'-3')** | **Destination** |
| --- | --- | --- |
| *GhLBD18-1-F* | GGATCCATGAGTTCAAATCCTAGTAACAG | *GhLBD18-1*gene cloning |
| *GhLBD18-1-R* | CTCGAGTCATTTAGAGAGCGATCGCGAGGGTG | *GhLBD18-1*gene cloning |
| *GhLBD18-1-RT-F* | TTTGAGGAGGAAATGTGTGCC | q RT-PCR |
| *GhLBD18-1-RT-R* | AAACCTTATGAACCGCTGCGA | q RT-PCR |
| *GhLBD16-1-F* | GGATCCATGGCATCAT CAAGCAGTAGTA | *GhLBD16-1*gene cloning |
| *GhLBD16-1-R* | CTCGAGTTAATTCCTCATCTAAGGGCA | *GhLBD16-1*gene cloning |
| *GhLBD16-1-RT-F* | CCTTTCTGGCACTGTTCCAAG | q RT-PCR |
| *GhLBD16-1-RT-R* | CAGGTTCACGCTATCACCACTG | q RT-PCR |
| *GhLBD16-2-F* | GGATCCATGGCATCCT CTGGCAC | *GhLBD16-2*gene cloning |
| *GhLBD16-2-R* | CTCGAGTCAGTTCCTCATCATTCT | *GhLBD16-2*gene cloning |
| *GhLBD16-2-RT-F* | CAATCTTTCTGGTGGTCCTTCC | q RT-PCR |
| *GhLBD16-2-RT-R* | TCCTCTCTGCTTTGTATTTCTTGC | q RT-PCR |
| *GhLBD29-F* | GGATCCATGACAGGTT CTGGTGCCC | *GhLBD29*gene cloning |
| *GhLBD29-R* | CTCGAGTCATGAATATTGCTGAGCA | *GhLBD29*gene cloning |
| *GhLBD29-RT-F* | ATGACAGGTTCTGGTGCCCCTTGT | q RT-PCR |
| *GhLBD29-RT-R* | ACAGGGAGGTGAGCAAGCAG | q RT-PCR |
| *GhLBD18-2-F* | GGATCCATGAGGGCAG CCAACAATG | *GhLBD18-2*gene cloning |
| *GhLBD18-2-R* | CTCGAGTTAGCCAGAAGTGGAAGGCC | *GhLBD18-2*gene cloning |
| *GhLBD18-2-RT-F* | CCACAACCGCAACAGACTTCA | q RT-PCR |
| *GhLBD18-2-RT-R* | GGGTGACATTGGGAGACGAAT | q RT-PCR |
